# Tuberculosis Infection in a Patient Treated With Nivolumab for Non-small Cell Lung Cancer: Case Report and Literature Review

**DOI:** 10.3389/fonc.2019.00659

**Published:** 2019-07-24

**Authors:** Ronwyn van Eeden, Bernardo L. Rapoport, Teresa Smit, Ronald Anderson

**Affiliations:** ^1^The Medical Oncology Centre of Rosebank, Johannesburg, South Africa; ^2^Department of Immunology, Faculty of Health Sciences, University of Pretoria, Pretoria, South Africa

**Keywords:** checkpoint inhibitors, immune reconstitution, non-small cell lung cancer, pulmonary infiltrates, tuberculosis

## Abstract

Nivolumab (PD-1 inhibitor) and other immune checkpoint inhibitors are used primarily to promote reactivation of anti-tumor immunity. However, due to their generalized immunorestorative properties, these agents may also trigger an unusual spectrum of side-effects termed immune-related adverse events. In the case of the lung, pulmonary infiltrates in patients treated with the anti-PD-1 inhibitors, nivolumab, or pembrolizumab, especially patients with non-small cell lung cancer, can result from immune-related pneumonitis, which, until fairly recently was believed to be of non-infective origin. This, in turn, may result in progression and pseudo-progression of disease. An increasing body of evidence has, however, identified pulmonary tuberculosis as an additional type of anti-PD-1 therapy-associated, immune-related adverse event, seemingly as a consequence of excessive reactivation of immune responsiveness to latent *Mycobacterium tuberculosis* infection. The current case report describes a 56-year old Caucasian female who presented with microbiologically-confirmed tuberculosis infection while on nivolumab therapy for non-small cell lung cancer. Notably, the patient, seemingly the first described from the African Continent, had not received immunosuppressive therapy prior to the diagnosis of tuberculosis.

## Background

Nivolumab, the focus of this case report, has been shown to have a significant overall survival benefit compared to docetaxel in the setting of second-line treatment of non-small cell lung cancer (NSCLC). The toxicity profile of nivolumab is also better than that of docetaxel, although the spectrum of side-effects varies greatly (1). Nivolumab is an immune checkpoint inhibitor, which is a fully humanized IgG4 monoclonal antibody that blocks programmed death cell protein-1 (PD-1), an inhibitory receptor that down-regulates the anti-tumor effector functions of T cells, while also interfering with the generation of immunological memory (2).

The side-effects of checkpoint inhibitors are referred to as immune-related adverse events (IrAEs) and are apparently related to over-activity of the immune system (3). One of these side-effects is pneumonitis, which has an incidence of <1%. Pneumonitis is more commonly seen with PD-1 and PD-L1 inhibitors than with CTLA-4 inhibitors (4). It presents clinically as shortness of breath and cough, while radiological examination reveals infiltrates, which typically resemble interstitial pneumonia, manifesting as reticular nodular infiltrates and ground glass opacities (4, 5). These non-specific features are, however, shared by a broad range of pulmonary disorders of both infective and non-infective origin, complicating differential diagnosis, often necessitating analysis of sputum, bronchoscopy with washings, or even biopsy (4–6). Thereafter, treatment of pneumonitis should be initiated without delay to lessen morbidity and mortality (4–6).

A small number (*n* = 9) of recent studies, spanning the period 2016–2019 and encompassing only 11 patients, has alerted oncologists practicing immune checkpoint inhibitor-based immunotherapy to the emerging threat of pulmonary tuberculosis (PTB) as a cause of pulmonary infiltrates, possibly mimicking pneumonitis as an IrAE (7–15). All of these reports, which are summarized in Table 1, involve administration of the PD-1-targeted monoclonal antibodies nivolumab (7 patients) or pembrolizumab (4 patients) to patients suffering from lung cancer (*n* = 5), malignancies of the upper respiratory tract (*n* = 2), advanced melanoma (*n* = 2), Hodgkin's lymphoma (*n* = 1) and Merkel cell carcinoma (*n* = 1) (7–15). With respect to demographics, the patients were either of Asian (*n* = 6) or Caucasian (*n* = 5) origin, aged 49–87 years and predominantly male (*n* = 10) (7–15). To our knowledge, there are no reports of similar cases associated with the use of CTLA-4 and PD-L1 inhibitors.

**Table 1:** Summary of case reports documenting development of acute pulmonary tuberculosis in cancer patients treated with PD-1 inhibitors.

**Patient(s)**		**Country of origin of report**	**Type of malignancy**	**PD-1 inhibitor**	**Outcome**	**References**
**Age**	**Gender**					
87	M	Singapore	Hodgkin's lymphoma	Pembrolizumab	Survived	(7)
72	M	Japan	NSCLC^*^	Nivolumab	Not reported	(8)
59	M	China	Stage 4 pulmonary adenocarcinoma	Nivolumab	Survived	(10)^+^
50	M	France	Metastatic melanoma	Pembrolizumab	Survived	(9)
64	M	France	NSCLC	Nivolumab	Died	(9)
65	F	China	Advanced melanoma	Pembrolizumab	Survived	(11)
56	M	Denmark	NSCLC	Nivolumab	Not reported	(12)
49	M	Taiwan	Stage 4 squamous cell carcinoma of hard palate	Nivolumab	Died	(13)
59	M	USA	Nasopharyngeal carcinoma	Nivolumab	Died	(14)
83	M	USA	Merkel cell carcinoma	Pembrolizumab	Survived	(14)
75	M	Japan	Lung adenocarcinoma	Nivolumab	Sruvived	(15)

* Non-small cell lung carcinoma;

+ patient with pericardial tamponade.

With the exception of the patient with Hodgkin's lymphoma, who had received two cycles of chlorambucil and prednisolone several months prior to administration of pembrolizumab (7), none of the other patients had received prior treatment with corticosteroids or other anti-inflammatory/immunosuppressive agents, specifically tumor necrosis factor-α-targeted monoclonal antibodies, which may predispose for active opportunistic infection with *M. tuberculosis* (8–15). Likewise, anti-PD-1 therapy-associated lymphopenia was excluded as a possible cause of acute PTB. This led the authors of these studies, as well as other commentators (16), to propose reactivation of immune responsiveness to latent *M. tuberculosis* bacilli and an accompanying severe inflammatory response as the probable cause of PD-1-related disease. This scenario is similar to TB-associated immune reconstitution syndrome (IRIS) associated with initiation of antiretroviral therapy of HIV-infected patients (17).

The current case report represents a meaningful addition to the current literature on the association of anti-PD-1 based monoclonal antibody therapy of advanced cancer with subsequent development of acute PTB, as it may be the first description of this type of IrAE originating from sub-Saharan Africa, a region with a particularly high prevalence of TB.

## Case Presentation

Written informed consent was obtained for the publication of this “Case Report.” The patient was a 56-year-old Caucasian female accountant who was initially diagnosed with NSCLC in 2013. She presented with coughing, weight loss and progressive shortness of breath. The patient had severe chronic obstructive airway disease as a result of a 30-pack year history.

The diagnosis was made on the basis of the findings of fine needle aspiration cytology and a confirmatory biopsy of a right-sided cervical lymph node. Histology showed a moderately differentiated adenocarcinoma with an immunohistochemical profile, which was consistent with that of lung cancer (CK7 Positive, CK 20 Negative, and TTF1 Positive). Her initial staging CT showed a large right-sided perihilar mass with significant left and right hilar nodes, sub-carinal and para-tracheal lymphadenopathy, as well as multiple satellite nodules in both lungs.

A metastatic work-up was requested. An abdominal CT scan and a bone scan showed no evidence of metastatic disease. At diagnosis, the patient had stage IV disease (T4N3M1a). The patient was given first line treatment of carboplatin and gemcitabine. She had a partial response to treatment with a 60% reduction in the size of tumor bulk according to RECIST (18). In early 2014, the patient presented with progressive dyspnoea and radiological evidence of cut-off obstruction of the bronchus. This event was assessed as disease progression. Treatment consisted of palliative radiation to the hilar mass.

**Figure 1:**
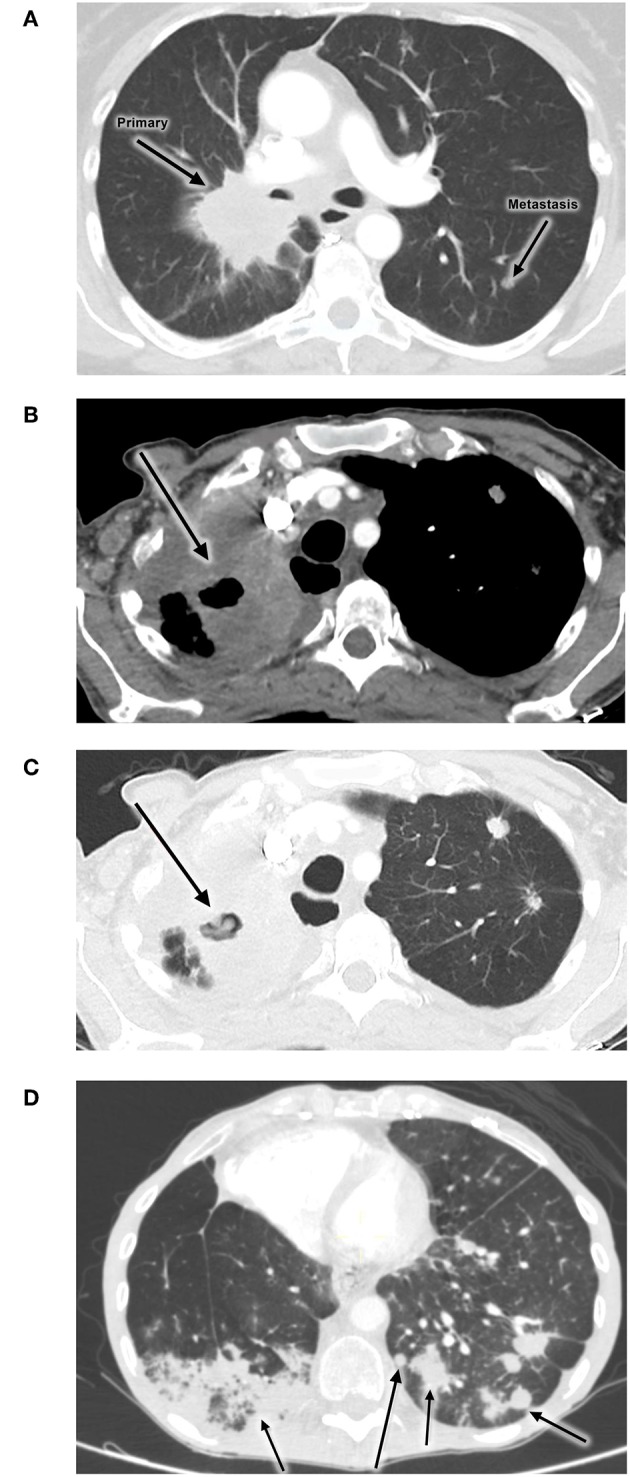
Images showing no evidence of PTB pre-treatment **(A)**, while **(B,C)** show development of PTB during administration of nivolumab. **(D)** shows a follow-up radiological investigation demonstrating cancer progression. **(A)** Initial disease progression (2 Apr 2014). **(B,C)** PTB infection during nivolumab treatment (22 Apr 2016). **(D)** Progression of disease (9 May 2016).

She subsequently received 6 cycles of single-agent pemetrexed when her symptoms became significantly worse (Figure 1A) up to mid-2015. Later in the year the patient's disease progressed and she received treatment with nivolumab as part of a South African expanded access program (EAP). This program was available for patients with NSCLC failing platinum-based chemotherapy in the first-line metastatic setting.

The patient experienced clinical improvement on nivolumab. However, in early 2016 she developed new symptoms of shortness of breath, cough and weight loss. A CT scan of the chest showed evidence of progressive disease, with new nodules bilaterally as well as new features of lymphangitis carcinomatosis. (Figures 1B,C). The new infiltrate was evident on the two different scans. An increasing right lung infiltration was noted, which was radiologically reported as either a secondary infection or disease progression. Pneumonitis was a differential diagnosis at the time.

The patient was hospitalized as her symptoms were severe. She was treated with intravenous corticosteroids and broad-spectrum antibiotics for possible bacterial infection. The infiltrate was atypical and sputum was tested for acid-fast bacilli (AFB) to check for PTB. It is relevant to note that this patient came from a good socioeconomic area with no known exposure to TB. Her HIV status was negative. Sputum test was positive for AFB with numerous organisms observed microscopically (>10 per field in 20 fields). The patient was referred to an infectious diseases specialist for PTB management (isoniazid, ethambutol, rifampicin and pyrazinamide). She improved clinically and was discharged on anti-TB medication and steroids tapered.

An attempt was made to restart nivolumab therapy, but the patient developed grade 2 diarrhea. After infectious causes were excluded, the diarrhea resolved with low dose oral corticosteroids. Shortly after that, the patient was readmitted for shortness of breath and respiratory symptoms and died in May 2016. Follow-up radiological investigation demonstrated cancer progression (Figure 1D).

## Discussion

The current case report, apparently the first originating from the African Continent, adds to the somewhat limited literature identifying PD-1-targeted immunotherapy of advanced cancer as a possible cause of apparent reactivation of latent *M. tuberculosis* infection, unrelated to prior administration of corticosteroids (19), possibly mimicking pneumonitis, a recognized IrAE.

Various causes of immunosuppression may predispose cancer patients to develop active or latent TB infection. Notwithstanding primary and secondary iatrogenic immunosuppression associated with stem cell transplantation for corrective therapy of hematological malignancies and that resulting from treatment with certain types of cytotoxic, anti-cancer chemotherapeutic agents, cancer *per se* may also result in secondary immunosuppression. This is certainly the case for those hematological malignancies which cause leukopenia, as well as for many types of solid tumor, which, due to their anatomic location cause obstruction and disruption of mechanical barriers (20). Cancer-associated immunosuppression may also be exacerbated by other factors such as immunosenescence, malnourishment and cigarette smoking (20, 21). With respect to development of PTB, the association of this infection with lung cancer and smoking is well-recognized (22), while associations of PTB with non-pulmonary malignancies, including Hodgkin's lymphoma, malignant melanoma (in males), as well as several other types of cancer have also been described (23, 24).

In the setting of anti-PD-1 therapy-associated pulmonary IrAEs, development of PTB, as well as some other types of microbial and viral infection, has, until recently, been attributed to opportunistic infection due to prolonged administration of corticosteroids to suppress pneumonitis (19). However, a report by Lee et al. in 2016 (7), subsequently confirmed by others (8–15), alerted clinical oncologists to the existence of alternative mechanisms of anti-PD-1-therapy-related development of acute PTB. In contrast to opportunistic infection with *M. tuberculosis*, these alternative mechanisms appear to involve triggering of exuberant, harmful pulmonary inflammatory responses due to reactivation of immune responsiveness to latent *M. tuberculosis* bacilli.

With respect to immunopathogenesis, Barber et al. in their recent description of two cases of PD-1 blockade-associated PTB, also reported that administration of the immune checkpoint inhibitor resulted in an increased prevalence of circulating *M. tuberculosis*-specific, interferon-γ (IFN-γ)-producing CD4 Th1 cells, but not Th17 cells, CD8 cells, regulatory T cells, or specific antibodies (14). These findings in humans are in agreement with several earlier studies in which CD4 T cell PD-1 gene knockout mice were used to investigate the role of this immune checkpoint inhibitor in the immunopathogenesis of acute, pulmonary *M. tuberculosis* infection (25–27). In these experimental animal studies, deficiency of PD-1 was found to result in excessive production of IFN-γ in the lungs of *M. tuberculosis*-infected mice, which, as opposed to controlling the infection, resulted in increased pulmonary bacterial loads, lung pathology and mortality, clearly underscoring a role for PD-1 in regulating harmful IFN-γ-mediated lung inflammation and dysfunction (25–27). However, as noted by Barber et al., further studies in the clinical setting are necessary to establish the exact mechanisms involved in PD-1-targeted therapy-related development of acute PTB (14).

Nevertheless, it is noteworthy in this context, that IFN-γ has been reported to suppress expression of the macrophage class-A scavenger receptor known as macrophage receptor with collagenous structure (MARCO), which promotes interaction of these cells with non-opsonized *M. tuberculosis* via binding to the bacterial cell wall glycolipid, trehalose 6, 6′-dimycolate (28–31). This type of interaction of the pathogen with MARCO promotes both phagocytosis and macrophage activation and is considered to be a major component of the host response to mycobacterial infection (29).

In conclusion, it is clear from the current and earlier reports that apparent reactivation of latent *M. tuberculosis infection* in many, but possibly not all cases (14), is a potential complication of PD-1-targeted immunotherapy of patients with advanced cancer. Where the index of suspicion may be high, this raises the issues of advance testing for latent TB and initiation of TB prophylaxis. In addition, as immune-based anti-cancer therapies become more affordable, these issues may become of particular relevance to developing countries where the prevalence of TB is high.

## Data Availability

No datasets were generated or analyzed for this study.

## Author Contributions

RvE, TS, and BR conceptualized the original case report. RA has critically reviewed the original manuscript and has contributed significantly to its restructuring and reviewing the literature.

### Conflict of Interest Statement

The authors declare that the research was conducted in the absence of any commercial or financial relationships that could be construed as a potential conflict of interest.
